# Columbia Program Digs Deeper into Arsenic Dilemma

**DOI:** 10.1289/ehp.113-a374

**Published:** 2005-06

**Authors:** M. Nathaniel Mead

The German idiom *verschlimmbesserung* refers to an intervention that is made with the best intentions to solve a problem but ends up worsening the situation or creating new problems. Past efforts to improve the drinking water supply of Bangladesh are a classic example. Three decades ago, the people of this poverty-stricken country got their drinking water primarily from surface sources, which were often contaminated with fecal pathogens that caused diarrhea, cholera, typhoid, and other life-threatening diseases. In 1971, the United Nations Children’s Fund launched a campaign to drill hand-pumped tubewells into the Ganges Delta alluvium. Hailed as a public health coup, the effort led to a plummet in waterborne microbial diseases throughout Bangladesh. But in the mid-1990s, local physicians noted a steep increase in the incidence of arsenicosis and other arsenic-related diseases—a trend subsequently linked to the drinking of tubewell water naturally rich in the metalloid element.

Now scientists at Columbia University have devised an approach that they believe could become part of an overall strategy to resolve the arsenic crisis in Bangladesh. Spearheaded by Alexander van Geen, a senior research scientist at the Lamont-Doherty Earth Observatory, Habibul Ahsan, an associate professor of epidemiology, and Joseph Graziano, associate dean of research at the Mailman School of Public Health, their proposed strategy includes the installation and monitoring of deep community wells in affected villages throughout Bangladesh. There is also a strong emphasis on training and organizing villagers at the community level to secure a source of safe water that is tailored to local needs.

The proposed strategy grew out of field work and a well survey conducted through the university’s NIEHS Superfund Basic Research Program, of which Graziano is director. The program was established in 2000 to study the bioavailability, health effects, and geochemistry of arsenic and lead. Projects to date have included bioavailability and geochemistry studies at four Superfund sites, epidemiologic and geochemistry studies of arsenic in drinking water in Bangladesh, and the development of practical remediation strategies for arsenic in wastewater and drinking water.

## Public Health Crisis

Today, 97% of the Bangladesh population drinks water from an estimated 10 million tubewells, according to research by van Geen and colleagues published in volume 81, issue 9 (2003) of the *Bulletin of the World Health Organization*. The contamination is most common in tubewells drawing groundwater from 10- to 100-meter depths in much of southern Bangladesh and, to a lesser extent, in northern areas along the Ganges River. A survey published in 2001 by the Bangladeshi Department of Public Health Engineering (DPHE) and the British Geological Survey (BGS) mapped out the variable extent to which different *upazilas* (subdistrict administrative units) were affected. The surveyors concluded that 35 million Bangladeshis are exposed to groundwater arsenic at concentrations exceeding the country’s standard of 50 micrograms per liter (μg/L), and 57 million are exposed to concentrations exceeding the World Health Organization (WHO) standard of 10 μg/L.

Prior to the inception of their investigations into chronic arsenic exposure in Bangladesh, the Columbia research team faced ethical review boards in both the United States and Bangladesh. “The Bangladesh committee on ethics in human research argued vehemently that we couldn’t just study the situation; we had to do something to reduce the population’s exposure to arsenic,” says Graziano. “This echoed the views of the institutional review board here in the States, and it has been our central credo ever since. Whatever our research findings might be, if we failed to lower the population’s exposure, we would fail, period.”

## Digging Deeper

In the spring of 2000, Graziano, van Geen, Ahsan, and others began a pilot study in Araihazar *upazila*, an area with a wide range of arsenic exposure. They used questionnaires to collect information on household water usage, awareness of arsenic-related risks, and preferences for remedial options should a well turn out to be unsafe. They used handheld Global Positioning System (GPS) receivers to map the location of each well. A few months later, the team began recruiting residents of this region into a prospective cohort study, and urine arsenic data were obtained for 12,000 residents, or about 17% of the study area’s population.

Nearly half the wells gave water with an arsenic content exceeding the national standard of 50 μg/L. Moreover, the team found that the distribution of arsenic in this region showed a high degree of spatial variability and was therefore difficult to predict. The proportion of safe wells varied greatly from one village to the next. But the high resolution of the GPS-based mapping enabled the Columbia team to clearly discern patterns in the spatial variability between the wells.

“We quickly realized that this spatial variability in high- and low-arsenic wells was our most valuable finding,” says van Geen. “Even though the highly variable nature of the [arsenic] distribution would complicate the intervention, it was clear that this high degree of spatial variability also presented an opportunity for remediation that needed to be more fully explored. For mitigation purposes, it seemed more important to know the proportion of unsafe wells in a particular village and [the variation of arsenic toxicity relative to depth] because this would be very different from the average value obtained for the entire *upazila*.”

Close to 90% of the Araihazar inhabitants, on average, were found to live within 100 meters of a safe well. Another promising finding was that 65% of households with an unsafe well had responded to the Columbia team’s testing and information dissemination by switching their usage to a nearby safe well. Within a few months of the testing, the mean urinary arsenic concentration among the 12,000 villagers tested previously had already significantly declined.

In 2001, the Columbia group installed seven community wells in villages where nearly all of the wells gave water with arsenic in excess of 50 μg/L. Two years later, 79% of study residents living within 150 meters of these community wells had switched to the community wells for drinking and cooking water. Their average urinary arsenic concentration dropped over this period from 204 to 91 μg/L, a figure approaching the 70 μg/L seen in residents of the same region who drank from low-arsenic wells.

Eventually the Columbia team charted the spatial scale of arsenic variability in 6,000 tubewells within a 25-square-kilometer area. In a report published in volume 80, issue 9 (2002) of the *Bulletin of the World Health Organization*, they concluded that well switching—the practice of sharing the safe wells that were frequently interspersed with unsafe wells—might be a viable temporary option in this area and possibly throughout Bangladesh. But they also cited anecdotal evidence suggesting that many villagers would not continue to fetch their water from a relatively distant well, especially one owned by another household (the vast majority of the wells surveyed were privately owned). In all likelihood, then, well switching was only a short-term solution, and one that might prove difficult to implement on a national scale.

## In Search of a Long-Term Solution

One long-term solution seemed to reside in the deep groundwater aquifers—those sometimes beyond the reach of tubewells—which were consistently low in arsenic. These aquifers, formed probably 40,000-plus years ago, offer a promising source of drinking water for the long term because no treatment and little maintenance is required for the wells, according to van Geen. “The geology of Bangladesh is now understood well enough to guarantee that a three-hundred-meter well in many parts of the country will tap into a low-arsenic aquifer, and in many parts of the country lesser depths will be equally safe,” he says. Fortunately, he adds, the vast majority of rural households reside within drilling distance of aquifers that are consistently low in arsenic.

The Columbia strategy focuses on creating a network of trained village workers, with an emphasis on community-based decision making. One person in each affected village would be provided with and trained to use a field kit for measuring arsenic, a handheld GPS receiver to determine each well’s position, and a handheld computer to enter field data. Groups of approximately 20 workers from different villages would then communicate this information digitally to a supervisor, who would be linked by wireless phone to a national support center to submit data for quality control and analysis. These collaborators would apply a simple decision tree to help village residents develop their plan for obtaining safe water, enabling them to determine the optimal depth and location of up to five deep community wells per village.

The private sector would likely play the major role in construction, maintenance, and possibly operation of the wells. The manpower and technical capability would come from well-drilling companies in Bangladesh and other countries. Nongovernmental organizations would serve as facilitators, building links between communities, local government, and local businesses. Funding would have to come from the government.

Graziano and van Geen predict that 100,000 wells could be installed for less than US$100 million (less than $1 per Bangladeshi citizen) and would enable the vast majority of households to be within a short walking distance of a safe community well. “If one considers the total costs, it is not a big price to pay, given the many benefits that would accrue to the public health and economy,” says Mushtaque Chowdhury, deputy executive director of the nongovernmental Bangladesh Rural Advancement Committee.

Moreover, a huge part of the plan’s cost-effectiveness derives from its target-specific nature. The Columbia team has demonstrated that tailoring the installation of community wells to local conditions halved the cost relative to what it would have been under a policy of blanket installations to a 300-meter depth. The cost would be reduced to a third if only the highest-risk areas are addressed initially, says Ahmed.

“By carefully considering the local geology, taking into account the variability in depth at which low-arsenic groundwater occurs, and precisely mapping this variability, the Columbia strategy enables us to pinpoint safe aquifers for the installation of community wells,” says Kazi Matin Ahmed, a professor of geology at the University of Dhaka and coauthor of the landmark BGS/DPHE report. “This is a more promising option than treating high-arsenic groundwater or microbe-contaminated surface water. The plan also emphasizes continued monitoring, which is currently lacking for the water supply wells in Bangladesh.” Ahmed advocates a national water quality surveillance program that would monitor levels not only of arsenic, but of lead, manganese, organic pollutants, and other contaminants of concern as well.

According to Graziano, the deep well strategy would easily achieve the WHO standard of 10 μg/L—an important point, given his team’s research in adults and children as well as other recent epidemiologic evidence indicating that the 50 μg/L guideline is not adequate to protect public health. Graziano asserts that the Bangladeshi standard reflects a somewhat arbitrary threshold, and that, over the long term, the WHO guideline should be followed whenever possible.

## One Plan of Many?

The Columbia plan has its fair share of critics, such as Harvard physics professor Richard Wilson, who says the approach overlooks many of the mitigation options proposed at the International Workshop on Arsenic Mitigation, organized by the WHO for the Bangladeshi government and held in Dhaka in January 2002. “Several alternative ways of obtaining pure water were suggested, each one appropriate for a different area, each with its advantages and disadvantages, and several now being successfully implemented,” says Wilson.

Wilson adds that all aspects of the Columbia strategy—or any other strategy—will require intensive community education and involvement, as well as continuous monitoring at the national level. This is the hardest problem, he says, and the one that has delayed all efforts so far and could make the strategy difficult to execute.

Wilson urges instead choosing from the list of alternative mitigation options developed at the January 2002 meeting, which include the use of dug wells, rainwater collection, and simple arsenic-removing techniques such as filtration at the household level.

Although these alternative strategies are technically feasible, Graziano and van Geen feel that most of them will never work over the long term in the dollar-a-day economy of Bangladesh. “Home filters require attention every day and will by definition run out of capacity at some point,” says Graziano. Rainwater collection, he notes, does appear to be a viable mitigation option in certain parts of the country and could therefore be considered an alternative or complement to the deep community well strategy.

Wilson voices another concern as well: that careless implementation of any strategy can be dangerous. “For example,” he says, “deep wells . . . [if] carelessly installed and not monitored, can lead to cross-contamination in some areas. If the deep aquifer becomes contaminated by the arsenic-laden aquifer, the strategy will have failed.”

Other experts disagree, to a point. “There is little danger for leakage along the outside of the well casing,” says Martin Stute, director of the Columbia program’s Research Core Laboratory for Hydrogeology. “The fine sediments of the Ganges–Brahmaputra Delta are closing in around any well installations very quickly. Indeed, the potential problem lies in breakage of the casing in the shallow aquifer due to shifting sediments.” Stute says periodic arsenic measurements can detect this problem, and if it occurs, the well should be filled with clays and redrilled. “However,” he says, “monitoring data collected so far indicate that this process is very unlikely to happen.”

In the meantime, education remains the key to change. In 2003, Columbia launched its Building Capacity to Reduce Arsenicosis in Bangladesh training program with funds from the John E. Fogarty International Center. Under this program, Bangladeshi pre- and postdoctoral students who are accepted into Columbia receive funding for two years of health, social, and Earth sciences training in the United States, then two years of field training in Bangladesh. Columbia faculty have also traveled to Bangladesh to conduct several short courses in environmental health, GIS technology, and geochemistry. Most recently, faculty have agreed to participate in the teaching of courses at a new school of public health in Dhaka.

Above all, Graziano says, any mitigation approach should be designed to rapidly reach the largest number of affected people. Only time and further research will tell whether the Columbia approach—or any other approach—can be effectively put into action in Bangladesh.

## Figures and Tables

**Figure:**
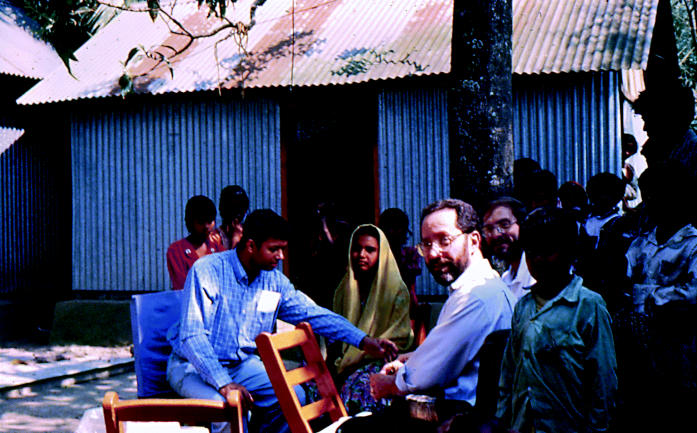
**The reason for the research.** Habibul Ahsan (left, in blue shirt), Joseph Graziano (center left, in glasses), and Paul Brandt-Rauf (center right, in glasses) of the Mailman School of Public Health meet with residents of Araihazar, Bangladesh.

**Figure:**
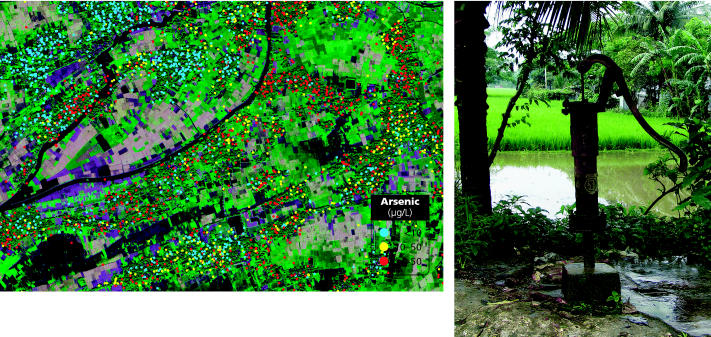
**High-tech search for low arsenic.** The Columbia project used GIS mapping (above) to survey arsenic concentrations in wells around Araihazar, Bangladesh. Safe wells are thusly labeled (right), and residents are encouraged to use them for drinking and cooking water.

